# Domestic

**Published:** 1841-03-20

**Authors:** 


### DOMESTIC.

We learn from a late number of the Bulletin of the Academy, that Tessier, the celebrated writer on agriculture and the diseases of grain, is dead at the advanced age of nearly a century. He was a member of the Royal Academy of Medicine; and his eulogium, as is usual in such cases, was read by the perpetual secretary, M. Pariset.

As the subject of diseases of plants and of animals is one which is more or less closely connected with medicine, either from the analogy which exists in the disorders of different classes of living beings, or from the diseases of the human race to which unwholesome food gives rise, we insert from the Farmer's Cabinet an interesting article on the rust of wheat, one of the most important diseases to which that plant is liable. It is written by a scientific gentleman of this city, who has devoted great attention to the study of agriculture. The analogy between this disease of wheat and the mode in which many disorders attack the human frame, is very apparent: and the physiological relations between plants and animals are probably not more closely connected than their diseased states. In the present instance the researches of the author of this essay on the rust of wheat led him to the study of the source of animal heat; and he has strong reason to believe the correct theory is that which ascribes the formation of heat to the conversion of the fluid blood into the solids of the body. The objections which have been urged against this theory are much less forcible when the analogy between plants and animals is kept in view; and we may, on a future occasion, be able to furnish some interesting developments upon this subject, which place many facts relative to it in a new point of view.

*Mildew, or meldew.*—After reading an essay in the last number of the Cabinet headed "Blight on Wheat," I was induced to read what numerous authors had written on the subject of mildew, rust, and blight; but on reflection, in regard to the *terms*, it was thought best to expunge the latter, as too general and indefinite to be applied to a specific form of disease, the character of which is well known to farmers, though the *cause* is not well understood. We say, familiarly, that any species of grain, fruit, &c., that fails in arriving at perfection, is *blighted*, without any reference to the form, character, or cause of the disease or accident, which has given rise to the failure.

The term *mildew*, or *meldew*, which I suspect was the original word, is formed of the word *mel*, which means honey, and *dew*, which needs no explanation; the combined term being, in plain English, *honeydew*, which, no doubt, took its designation from a sweet substance found on the surface of the wheat after it became diseased, and which is now believed to be the excrement of very small insects, which attack the plant after the hand of death has come upon it, and decomposition has commenced. The term *rust* needs no explanation to the farmer who has once suffered by this form of disease in his grain crop. These two terms indicate forms of disease, both of which, it is believed, proceed from one common cause, and are only slight modifications of the same disastrous malady.

Some of the authors which have been examined, ascribe the disease to great heat, some to hoar frost or cold after great heat, electricity, diseased seed, disease of the root of the plants, the presence of the barbarry bush, fungi, insects, wind from particular quarters, and various other causes, all equally unsatisfactory.

It is pleasant and satisfactory to be able to trace the diseases, both of animals and vegetables, to their true cause, even if we should not be enabled to prescribe a remedy for them; and upon reflection in regard to the various circumstances connected with the mildew in grain-crops, some thoughts have arisen and have matured into opinions, which I will throw into the common stock, that farmers may judge of them, and see how far they agree with their own experience and observation.

The conditions under which it is believed that wheat arrives at the greatest perfection are, a cool season and a reasonably dry atmosphere at the time of the filling and maturation of the grain; it being assumed that the soil is sufficiently moist to furnish the requisite nutriment for a healthy state of the plants.

The summer of 1816 was of this description, and the wheat of that year was of extraordinary weight and fineness; and what was then considered of equal importance, the crop, when brought to market, sold for three dollars per bushel of 60 lbs., a price it has never since commanded. A worthy farmer, residing in Montgomery county, had that season an average of 32 bushels and 8 lbs. per acre for his whole crop, and sold it for 3 dollars per bushel, being a little more than 96 dollars per acre, and the farm on which it grew cost him 24 dollars per acre some twenty years previous.

The circumstances, which are supposed to be always present in a greater or less degree when the crop becomes diseased with *mildew*, are the following, viz.: The atmosphere saturated with moisture, in the form of fog or otherwise, a high temperature of the air, and scarcely a breath of wind stirring, the latter being a necessary consequence of the former conditions, because, if the wind was active, the moisture would be dissipated and the heat rendered less sensible. In fact, such weather as is familiarly known by the term hot and muggy, and which sometimes relaxes the human system to that degree that it seems as if it was on the point of dissolving; the reason being, that the air is so saturated with moisture that it refuses to take up the insensible perspiration from the surface of the human body, and the heat, which would necessarily go along with it, remains pent up within us, without a possibility of escape, to our great suffering and discomfort.

There are other conditions of the crop, which render the above circumstances more certainly fatal; but it is doubted whether *alone* they would be much detrimental in producing mildew, such as a very succulent, vigorous growth, produced by manuring too highly, &c.

Now for the *theory*; the word I don't like much, because farmers in general complain so much about theories; but have them we must, while men continue to *think;* for if we think about facts, we are constantly forming theories, in spite of our wishes to the contrary; but theories are only bad or wrong when they don't explain facts, and of this every man must judge for himself. If theories are true, and give sound reasons for things, they are good theories; if not, they are bad, and should be discarded without ceremony.

All plants derive their nutriment from the earth, and take it up in a state of very dilute solution in water; this is elaborated in their organs, in a way incomprehensible to us, and deposited where it is needed, to promote the growth and expansion of the plant, and enable it to perform the functions designed in its creation; and the great object would seem to be, to cause it to perfect its seed, and continue its species. When this deposit of nutriment is made, the water, which was its vehicle of conveyance, is thrown out of the plant as excrementitious by proper exhaling vessels, and is dispersed, in the form of vapour, in the atmosphere, and the vessels of circulation, which are in continual action, introduce continued supplies of similar nutriment duly prepared for deposit, and throw off the water as before, so that there is never a vacuum in the plant.

This process is in continual action till the plant is perfected, and the quantity of water thrown off during the progress of vegetation is almost incredible, as would appear by some very accurate experiments made to indicate the amount. Of 15 parts of water taken up by some plants, 13 are transpired, and of the lowest on the scale, of 5 parts taken up 4 are discharged by exhaling vessels.

Wheat and other plants, when they have acquired their full growth, commence the interesting business of perfecting their seeds, in order to perpetuate their kind; and then nature brings all her powers into requisition to effect this remarkable process. The vessels of the plant are distended with the proper fluid making its way to the seed-vessel, which has been duly prepared to receive it, which is there deposited in the form a milky juice, when the water, which has been the vehicle for its conveyance, is discharged through exhaling vessels into the atmosphere, and another supply from the same source is constantly in the rear, to be disposed of in a similar manner; and so the process goes on—provided there is no unfortunate interruption from external causes—till each grain is filled with farina, when, the great work being completed, the circulation ceases to be carried on, and the whole is dried and hardened for preservation.

When this process is going forward, it will be perceived that a vast proportional quantity of water must be constantly discharged into the atmosphere, otherwise, space would not exist in the hull of the grain for additional supplies of the diluted nutriment, which is continually arriving at its destined depository; but should the atmosphere at this critical period unfortunately be *saturated* or *surcharged* with *moisture*, as has been before hinted, it will be unable to take up and carry away that which the grain must necessarily part with, and which is now an incumbrance to it, in order to make room for an additional supply of the fluid which would increase the deposit of farina. This inability of the air to take up an additional load of moisture under the circumstances of its previous *saturation*, prevents it also from carrying off the heat from the wheat, so that the temperature of the whole plant is increased much above the proper standard of its healthy action; for the temperature of plants that transpire moisture freely is constantly kept many degrees cooler than the surrounding atmosphere, or bodies destitute of vital action. This retention of excrementitious moisture suspends the circulation, for it can't move unless it can get vent, and that and the expansion occasioned by increase of temperature produce congestion, and burst the vessels of circulation, and discharge their contents into the cellular texture, destroy the vitality of the plant, and leave the hull only partially filled with farina. Heat, air and moisture, the agents of putrefaction, being present, decomposition begins, and the surface of the plant soon displays signs of decay. This destructive process first shows itself in the smallest part of the stalk, near the head, which is of most recent formation, and consequeutly most succulent and tender, and most liable to rupture. The rust is probably occasioned by the heat and internal pressure enlarging the pores of exhalation and discharging the sap on to the surface of the stalk, and when evaporation carries off the moistnre, the residuum displays itself somewhat like the rust of iron. After the rupture and discharge of the sap vessels, the surface of the plant is covered with mucus which is adhesive, and this will account for the seeds of fungi, which are supposed to be floating in abundance in the atmosphere, taking root and vegetating in the decaying structure; and hence the supposition, that fungi are the *cause of mildew.*

The presence of animalcula may be accounted for on the same principle, for nature is ever economical, and wherever animal or vegetable substances are in the progress of decay, mouths are found ever ready to convert dead matter into food for living things, so as to perpetuate the largest possible amount of animated existence. On the death of the plant, the tender succulent fibres of the roots immediately decay, and on drawing them from the ground, the appearance of them has led many to suppose that they had thus discovered the true *cause* of the disease of the plant, when, in fact, it was only the *effect* of its previous dissolution.

It may be objected, that if the mildew is the result of a general saturation of the atmosphere with moisture at a particular period, that all wheat should be equally injured by it; when the fact is well known, that of contiguous fields one will be destroyed and the other remain uninjured. This apparent contradiction is accounted for by the uninjured wheat being more forward in filling than the other, for the whole mischief takes place in a few days, perhaps a few hours; and where the grain has received the requisite supply of nutriment the circulation is diminished or suspended, and there is no danger of injury from the canals breaking their banks and overflowing.

The grain in the same inclosure is often partially injured; in low and damp spots, or where it is lodged, for want of the requisite circulation of the air to dissipate the moisture, the injury is great from mildew; when in the higher and drier parts parts of the same field, it is protected from injury, by a freer circulation preventing the mischief by carrying off the excess of moisture and promoting a healthy transpiration from the plants.

The elasticity of plants favours their being put in motion by the wind, and this no doubt increases the circulation of the sap, in the same way that motion in animals promotes the circulation of the blood; and, at the same time, it favours the evaporation of moisture and promotes a healthy condition. On this principle, it is supposed that good may arise from passing a rope extended across the ridges of wheat in in such a manner as to communicate motion to the stalks, and in some measure dissipate the excess of moisture on the principle of *fanning*, as has been recommended by some writers on the subject of mildew. But perhaps the most effectual plan of guarding against this fatal disease would be to seek for and sow only the earliest varieties of wheat, which fill before the season arrives most likely to be accompanied by that condition which is the cause of the injury.

AGRICOLA.

### HEALTH OF THE CITY.

INTERMENTS *in the City and Liberties of Philadelphia, from the* 6*th to the* 13*th of March*, 1841.

| Diseases. | Adults. | Children. | Diseases. | Adults. | Children. |
|---|---|---|---|---|---|
| Asthma, | 2 | 0 | *Brought forward*, | 39 | 51 |
| Abscess of lungs, | 0 | 1 | Intemperance and | | |
| —— kidneys, | 1 | 0 | exposure, | 1 | 0 |
| —— thigh, | 1 | 0 | Inanition, | 0 | 1 |
| Apoplexy, | 1 | 0 | Marasmus, | 0 | 1 |
| Burn, | 0 | 1 | Measles, | 0 | 4 |
| Cancer, | 1 | 1 | Mania a Potu, | 1 | 0 |
| Caries of spine, | 0 | 1 | Old age, | 1 | 0 |
| Croup, | 0 | 2 | Palsy, | 2 | 0 |
| Cong'n of lungs | 0 | 4 | Pleurisy, | 1 | 0 |
| Consumption of | | | Scrofula, | 0 | 2 |
| the lungs, | 14 | 8 | Small pox, | 3 | 5 |
| Convulsions, | 0 | 7 | Still-born, | 0 | 5 |
| Dropsy, | 3 | 0 | Suicide, | 1 | 0 |
| —— abdominal, | 1 | 0 | Tetanus, | 0 | 1 |
| —— head, | 0 | 6 | Unknown, | 1 | 0 |
| Dis'se of the brain, | 0 | 1 | Worms, | 0 | 1 |
| —— heart, | 0 | 1 | | — | — |
| —— stomach and | | | Total, 121— | 50 | 71 |
| bowels, | 1 | 0 | | | |
| Debility, | 0 | 1 | Of the above, there | | |
| Erysipelas, | 0 | 1 | were under 1 year | | 31 |
| Fever, typhus, | 2 | 0 | From 1 to 2 | | 16 |
| —— typhoid, | 1 | 0 | 2 to 5 | | 8 |
| Hooping-cough, | 0 | 1 | 5 to 10 | | 9 |
| Hernia strangul'd | 1 | 0 | 10 to 15 | | 3 |
| Influenza, | 1 | 0 | 15 to 20 | | 6 |
| Inflammation of | | | 20 to 30 | | 12 |
| the brain, | 0 | 3 | 30 to 40 | | 7 |
| —— bronchi, | 0 | 6 | 40 to 50 | | 9 |
| —— lungs, | 6 | 4 | 50 to 60 | | 7 |
| —— stomach and | | | 60 to 70 | | 8 |
| bowels, | 1 | 1 | 70 to 80 | | 5 |
| —— liver, | 1 | 0 | 80 to 90 | | 0 |
| —— peritonæum, | 1 | 1 | 90 to 100 | | 0 |
| | — | — | | | —— |
| *Carried forward*, | 39 | 51 | Total, | | 121 |

In the above are included 21 people of colour, and 4 interments from the alms-house.

